# Decellularized Organ-Derived Scaffold Is a Promising Carrier for Human Induced Pluripotent Stem Cells-Derived Hepatocytes

**DOI:** 10.3390/cells11081258

**Published:** 2022-04-07

**Authors:** Hideaki Kojima, Hiroshi Yagi, Hiroko Kushige, Yukiko Toda, Kazuo Takayama, Shinako Masuda, Toshinori Morisaku, Tomonori Tsuchida, Kohei Kuroda, Kazuya Hirukawa, Jumpei Inui, Kotaro Nishi, Yutaka Nakano, Masayuki Tanaka, Shutaro Hori, Yasushi Hasegawa, Yuta Abe, Minoru Kitago, Shungo Adachi, Masatoshi Tomi, Katsuhisa Matsuura, Hiroyuki Mizuguchi, Yuko Kitagawa

**Affiliations:** 1 Department of Surgery, Keio University School of Medicine, Shinanomachi 35, Shinjuku 162-8666, Japan; hide.cat.caprichoso@keio.jp (H.K.); hiroko.kushige@keio.jp (H.K.); morisaku@keio.jp (T.M.); tsuchida_t@keio.jp (T.T.); k.kuroda@cc.miyazaki-u.ac.jp (K.K.); kazuya.hirukawa@gmail.com (K.H.); nishi24@keio.jp (K.N.); yutaka.nakano.9833@gmail.com (Y.N.); masa_psg@hotmail.com (M.T.); shutaro.hori@gmail.com (S.H.); hasegawayas@yahoo.co.jp (Y.H.); abey3666@gmail.com (Y.A.); dragonpegasus@keio.jp (M.K.); kitagawa@a3.keio.jp (Y.K.); 2 Laboratory of Biochemistry and Molecular Biology, Graduate School of Pharmaceutical Sciences, Osaka University, Suita 565-0871, Japan; toba-y@phs.osaka-u.ac.jp (Y.T.); takayama@phs.osaka-u.ac.jp (K.T.); inui-j@phs.osaka-u.ac.jp (J.I.); mizuguch@phs.osaka-u.ac.jp (H.M.); 3 Institute of Advanced BioMedical Engineering and Science, Tokyo Women’s Medical University, Shinjuku 162-8666, Japan; masuda-aoki.shinako@twmu.ac.jp (S.M.); matsuura.katsuhisa@twmu.ac.jp (K.M.); 4 Molecular Profiling Research Center for Drug Discovery, National Institute of Advanced Industrial Science and Technology, Aomi 2-4-7, Koto 162-8666, Japan; s.adachi@aist.go.jp; 5 Division of Pharmaceutics, Faculty of Pharmacy, Keio University, Shibakoen 1-5-30, Minato 162-8666, Japan; tomi-ms@pha.keio.ac.jp; 6 Laboratory of Hepatocyte Regulation, National Institutes of Biomedical Innovation, Health and Nutrition, Ibaraki 567-0085, Japan; 7 Global Center for Medical Engineering and Informatics, Osaka University, Suita, Osaka 565-0871, Japan; 8 Integrated Frontier Research for Medical Science Division, Institute for Open and Transdisciplinary Research Initiatives, Osaka University, Suita 565-0871, Japan

**Keywords:** human iPSCs, decellularization, organ-derived scaffold, extracellular matrix, microminiature pig, xeno-implantation, xeno-transplantation

## Abstract

Human induced pluripotent stem cells (hiPSCs) are a promising cell source for elucidating disease pathology and therapy. The mass supply of hiPSC-derived cells is technically feasible. Carriers that can contain a large number of hiPSC-derived cells and evaluate their functions in vivo-like environments will become increasingly important for understanding disease pathogenesis or treating end-stage organ failure. hiPSC-derived hepatocyte-like cells (hiPSC-HLCs; 5 × 10^8^) were seeded into decellularized organ-derived scaffolds under circumfusion culture. The scaffolds were implanted into immunodeficient microminiature pigs to examine their applicability in vivo. The seeded hiPSC-HLCs demonstrated increased albumin secretion and up-regulated cytochrome P450 activities compared with those in standard two-dimensional culture conditions. Moreover, they showed long-term survival accompanied by neovascularization in vivo. The decellularized organ-derived scaffold is a promising carrier for hiPSC-derived cells for ex vivo and in vivo use and is an essential platform for regenerative medicine and research.

## 1. Introduction

Recent medical advances have improved the quality of life and prognosis of patients with end-stage organ failure, such as cirrhosis [1]. However, the gradual worsening of such organ failure is inevitable, and organ transplantation is the only curative treatment option for patients. Thus, the importance of research on regenerative medicine has increased to understand the pathogenesis of diseases and the compensation of organ functions. At present, induced pluripotent stem cells (iPSCs) are a promising cell source for regenerative medicine, owing to their pluripotency, self-renewal, and ability to construct patient-specific cell lines. Moreover, stem cell banking mostly relies upon iPSCs; >80% of registered stem cell lines are derived from iPSCs [2]. While the production of a large number of iPSC-derived cells is feasible for some cell types, such as hepatocytes or cardiomyocytes, there is an emerging demand for a carrier of these cells in in vivo-like environments, or direct use of a large number of iPSC-derived cells are required.

IPSC-derived cells have been explored for medical engineering. Nagamoto et al. reported on the amelioration of lethal acute liver injury in mice by transplanting sheets of human iPSC-derived hepatocyte-like cells (hiPSC-HLCs) [3]. A liver model that reproduces the structure of a liver lobule was developed by bioprinting hydrogel-embedded hiPSC-derived liver progenitor cells [4]. The biggest challenge in cell sheet engineering and bioprinting technology is the construction of mass 3D structures. Transplanted cells are generally maintained through the passive diffusion of oxygen and nutrients in these bioengineered systems. However, passive diffusion only has a limited capacity to maintain the engrafted cells. Thus, it is difficult to supply oxygen and nutrients to a large number of iPSC-derived cells embedded into sheets or printed gels with thicknesses exceeding the limit of cellular viability. In addition, a microenvironment similar to that in vivo is necessary to maintain the phenotypes and functions of iPSC-derived cells robustly.

A possible solution to this challenge is the use of organ-derived scaffolds obtained via decellularization techniques. Decellularization of tissues, which is achieved by chemically or physically removing all viable cells from native tissues [5], has developed to retain 3D structures, such as extracellular matrices (ECMs) and vascular networks, for advanced tissue culture and organ engineering. The organ-derived scaffolds allow retention of a large number of cells by continuous perfusion of cell culture media through preserved vascular structures or through the supply of oxygen and nutrient-rich blood flow in vivo use [6]. They form a cellular niche that contains growth factors and various signaling molecules such as proteoglycans, glycosaminoglycans, and glycoproteins. Cell viability, maturation, and differentiation are regulated through the interaction between ECMs and cells, mediated by these bioactive molecules [7,8]. A close relationship between cell phenotypes and ECMs is evident because associated cell-ECM interactions induce pathological degeneration of cells, resulting in the functional decline of tissues or organs [9,10]. Thus, it is expected that decellularized organ-derived scaffolds are a promising carrier for iPSC-derived cells.

Cell seeding into decellularized organ-derived scaffolds has been realized in a variety of organs and cell types, including liver [11], pancreas [12], and heart [13]. Recently, a rat liver-derived decellularized scaffold filled with iPSC-derived liver-constituent cells was xeno-transplanted into rats, indicating the possibility of an artificial liver constructed by the combination of decellularized organ-derived scaffolds and hiPSC-derived cells [14]. The use of a large number of iPS cells in humans is essential for the further development of clinical applications. However, studies on organ-derived scaffolds using iPSC-derived cells have not progressed considerably due to the requirement of combined multiple advanced techniques: mass culture technology of iPSC-derived cells, decellularization technology to obtain the scaffolds, advanced surgical transplantation technology, and postoperative management. In addition, the technology to induce all cell lines that constitute single organs from iPSCs has not been established yet. Thus, we used hepatocytes, which are the primary cells that account for 80% of liver-constituent cells and for which mass culture and differentiation induction techniques from iPSCs have been established [15], to evaluate the feasibility of clinical application of organ-derived scaffolds. In the liver, blood flow from the portal vein throughout the central vein nourishes and maintains the hepatocytes; in addition, it regulates hepatocyte phenotypes and metabolic functions by creating an oxygen concentration gradient [16]. Therefore, a bioengineered liver requires structural features that allow broad and uniform perfusion of culture medium; this would facilitate shear stress and physiological oxygen gradients to the engrafted cells. In addition, it is known that ECM proteins can modulate the capacity of albumin synthesis and liver-specific gene expression of hepatocytes [17]. Indeed, decellularized liver scaffolds preserve growth factors essential for hepatocyte maturation, such as HGF, bFGF, VEGF, and IGF-1 [18,19,20]. Therefore, it is reasonable to apply decellularized liver scaffold for hepatocytes from both structural and biochemical perspectives.

In this study, we used an organ-derived scaffold as a carrier of a large number of hiPSC-HLCs and demonstrated its efficacy in ex vivo evaluations in combination with circumfusion culture via the vascular network preserved in the scaffold. The possibility of its in vivo application, such as transplantable engineered organs, was also examined.

## 2. Materials and Methods

### 2.1. Animals

Göttingen minipigs (Oriental Yeast Co., Ltd., Tokyo, Japan) weighing approximately 15 kg were procured as donors of liver-derived scaffolds. Female thymectomized microminiature pigs (Fuji Micra Inc., Shizuoka, Japan) weighing 12–15 kg were used as recipients of hiPSC-HLC-recellularized liver scaffolds. The recipient pigs were immunosuppressive models that underwent thymectomy immediately after birth to inhibit T cell maturation [21]. A combination of retrorsine administration and partial hepatectomy was performed on recipient pigs to induce proliferation of implanted or transplanted hiPSC-HLCs. Intraperitoneal retrorsine (20 mg/kg; Sigma-Aldrich Co., St. Louis, MO, USA) was administered 42 and 28 d prior to transplantation, and a 60% hepatectomy was performed at the time of surgery, as previously reported [22]. Three micro-miniature pigs each were used for the implantation under the renal capsule and the transplantation via vascular anastomosis. The animals were handled according to the guidelines for the use of laboratory animals established by the Ministry of Education, Culture, Sports, Science, and Technology, Japan.

### 2.2. Preparation of the Porcine Liver Scaffold for Recellularization

The pig was anesthetized by intramuscular injection of 0.1 mg midazolam (Sandoz Ltd., Tokyo, Japan) and 0.02 mg/kg medetomidine (Orion Co., Turku, Finland). Thereafter, it was intubated and connected to a mechanical ventilation system with continuous inhalation of 2% isoflurane throughout the operation. Systemic heparinization was performed with an intravenous injection of 2000-U heparin. Following the incision of the diaphragm, the inferior vena cava (IVC) above the liver was dissected, and the pig was euthanized with the release of blood into the chest cavity. Following the quick dissection of the hepatic artery, portal vein (PV), and inferior vena cava, the whole liver was procured. An 18 Fr tube (Yamato Scientific Co., Ltd., Tokyo, Japan) was inserted into the portal vein and IVC of the procured liver, and 1000 mL saline was used for irrigation to remove residual blood. The process of decellularization of the liver is shown in Figure S1. The liver was stored at −80 °C until decellularization. Prior to decellularization, samples were gradually thawed for 48 h at 4 °C. Phosphate-buffered saline (PBS; FUJIFILM Wako Pure Chemical Corp., Osaka, Japan) was perfused through the PV at a rate of 70 mL/h using a liquid transfer pump (Masterflex L/S with an Easy Load Pump Head III; Yamato Scientific Co., Ltd., Tokyo, Japan) until the drainage fluid was transparent. The following day, 0.5% sodium dodecyl sulfate (SDS; Fujifilm Wako Pure Chemical Corp., Osaka, Japan) was perfused at a rate of 70 mL/h. The flow rate was gradually increased to 180 mL/h according to the progress of decellularization. Following 12 h of flushing with SDS, the scaffold was washed thoroughly with PBS until the effluent was not visually foamy. Thereafter, the scaffold was stored overnight at 4 °C. On the third day, 0.5% Triton X-100 (Sigma-Aldrich Co., St. Louis, MO, USA), 0.05% ethylene glycol-bis (β-aminoethyl ether)-N,N,N′,N′-tetraacetic acid (Dojinkagaku, Ltd., Tokyo, Japan), and 2 mmol 3-((3-cholamidopropyl) dimethylammonio)-1-propanesulfonate (CHAPS; Dojinkagaku, Ltd., Tokyo, Japan) were refluxed into the scaffold in the same manner for further decellularization, and the scaffold was subsequently maintained overnight at 4 °C after washing with PBS. On the fourth day, the decellularized whole liver was divided into two parts using a stapler (ECHELON FLEX™ ENDOPATH; Johnson & Johnson K.K., Tokyo, Japan), and the lateral right lobe was used. Following confirmation of the absence of leakage of fluid injected from the PV out of the vessel, the scaffold was immersed in PBS containing antibiotic-antimycotic 1 × (Thermo Fisher Scientific K.K., Tokyo, Japan), and subsequently immersed in 1% gentamicin (Nacalai Tesque Co., Ltd., Tokyo, Japan). The scaffold was sterilized with 25 kGy of γ-ray (RADIA INDUSTRY Co., Ltd., Gunma, Japan) and stored at 4 °C until recellularization.

### 2.3. Scanning Electron Microscopy (SEM)

Specimens of native and decellularized livers were fixed in 2.5% glutaraldehyde (30 mM 4-(2-hydroxyethyl)-1-piperazineethanesulfonic acid (HEPES) buffer) for 24 h, and subsequently stained with 1% tannic acid (30 mM HEPES buffer). Thereafter, specimens were fixed in 1% osmium tetroxide (30 mM HEPES buffer) for 2 h. The samples were dehydrated with 50, 70, 80, 90, and 100% ethanol for 30 min. Following the replacement of ethanol with *t*-butyl alcohol, specimens were lyophilized. The dried specimens were coated using an osmium coater and visualized using an SU6600 low-vacuum analytical scanning electron microscope (Hitachi High-Technologies Co., Tokyo, Japan).

### 2.4. Proteomic Assay

The decellularized liver tissue was cut into 10 mg pieces in the hepatic parenchyma, vein, and portal regions. Each piece was digested and peptised in a solution of 0.001% trypsin, 10 μM Tris-HCl, 0.005% n-octyl glucopyranoside, and 0.7 M guanidine hydrochloride (pH 8.8) for 12 h. Thereafter, they were reduced with 5 mM tris(2-carboxyethyl) phosphine for 30 min at 65 °C. Following alkylation with 10 mM iodoacetamide for 30 min at 25 °C, they were washed with a C18 monospin column (GL Science K.K., Tokyo, Japan). Purified peptides (500 ng) were separated using a C18 separation column (Nikkyo Technos Co. Ltd., Tokyo, Japan) and analyzed on an Easy-nLC 1200 coupled with a Q-Exactive HF-X instrument (Thermo Fisher Scientific K.K., Tokyo, Japan). Analysis was performed in data-dependent acquisition mode, where the top 25 recorded MS spectra between 380 and 1500 m/z were selected. Survey scans were acquired at a resolution of 60,000 at m/z 200, and MS/MS resolution was set to 15000 at m/z 200. All MS/MS spectra were searched against the protein sequences of the *Sus scrofa* (NCBI: txid9823) protein database using Proteome Discoverer 2.2 (Thermo Fisher Scientific K.K., Tokyo, Japan) equipped with the SEQUEST search engine. Gene ontology (GO) annotation enrichment analysis and Kyoto Encyclopedia of Genes Genomes (KEGG) pathway analysis using DAVID (Database for Annotation Visualization and Integrated Discovery) version 6.7 (https://david.ncifcrf.gov/ accessed on 15 July 2021) were performed to identify statistically significant biological themes and functional groups (*p* < 0.05).

### 2.5. DNA Quantification

Four samples, each of native and decellularized liver tissues, were used for DNA quantification (biological replicate). Samples were subjected to three technical replicates for each measurement, and the average value was used. DNA in the native and decellularized liver tissues was extracted using Trizol (Invitrogen Inc., Carlsbad, CA, USA), according to the manufacturer’s protocol. Briefly, samples weighing 80−100 mg were homogenized in 1 mL of Trizol using a Micro Smash MS-100 (TOMY Ltd., Tokyo, Japan). Homogenate was transferred to collection tubes, and 200 μL of chloroform was added, followed by centrifugation at 12000× *g* for 15 min at 4 °C. The supernatant was discarded, and 300 μL of 100% ethanol was added to the pellet and incubated at room temperature for 3 min. The DNA pellet was obtained by centrifugation at 2000× *g* for 5 min. It was washed twice with 0.1 M citrate buffer and resuspended with 1.5 mL of 75% ethanol. After 20 min of incubation, the sample was centrifuged at 2000× *g* for 5 min at 4 °C. The supernatant was removed, the pellet was dried for 15 min and then dissolved in 8 mM NaOH; the number of nucleic acids were quantified using NanoDrop One^C^ (Thermo Fisher Scientific K.K., Tokyo, Japan). The amount of DNA was normalized to the initial dry weight of the samples. DNA from the recellularized scaffolds and the cells cultured in vitro was extracted similarly. In the extraction from the cultured cells, the homogenization step was skipped.

### 2.6. Preparation of hiPSC-HLCs

The hiPSC, 1231A3 (gifted by Dr. S. Yamanaka (CiRA, Kyoto University); Number HPS0381), was maintained on 1 μg/cm^2^ recombinant human laminin 511 E8 fragments (iMatrix-511, Nippi, Tokyo, Japan) in StemFit AK02N medium (Ajinomoto, Co., Ltd., Tokyo, Japan). To passage hiPSCs, near-confluent hiPS cell colonies were treated with TrypLE Select Enzyme (Thermo Fisher Scientific K.K., Tokyo, Japan) for 3 min at 37 °C. Following centrifugation, hiPSCs were seeded at 5 × 10^4^ cells/cm^2^ onto iMatrix-511 and were subcultured every 6 d. Prior to the initiation of hepatocyte differentiation, hiPSCs were dissociated into single cells using TrypLE Select Enzyme and plated onto Matrigel-coated dishes. These cells were cultured in StemFit AK02N medium for 2–3 d. Induction of differentiation into definitive endoderm cells, hepatoblast-like cells, and hepatocyte-like cells was performed based on our previous reports with minor modifications [15]. Briefly, for definitive endoderm differentiation, hiPSCs were cultured for 4 d in Roswell Park Memorial Institute (RPMI) 1640 medium (Sigma-Aldrich, Co., St. Louis, MO, USA) containing 100 ng/mL activin A (R&D Systems, Inc., Minneapolis, MN, USA), 1 × GlutaMAX, and 1 × B27 Supplement Minus Vitamin A (Thermo Fisher Scientific K.K., Tokyo, Japan). To induce hepatoblast-like cells, definitive endoderm cells were cultured for 5 d in RPMI 1640 medium (Sigma-Aldrich Co., St. Louis, MO, USA) containing 20 ng/mL bone morphogenetic protein 4 (BMP4; R&D Systems, Inc., Minneapolis, MN, USA), 20 ng/mL fibroblast growth factor 4 (FGF4; R&D Systems, Inc., Minneapolis, MN, USA), 1× GlutaMAX, and 1× B27 Supplement Minus Vitamin A. To perform hepatic differentiation, hepatoblast-like cells were cultured for 5 d in RPMI1640 medium (Sigma-Aldrich Co., St. Louis, MO, USA) containing 20 ng/mL hepatocyte growth factor, 1× GlutaMAX, and 1× B27 Supplement Minus Vitamin A. Finally, hepatoblast-like cells were cultured for 11 d in a hepatocyte culture medium (HCM, Lonza, Basel, Switzerland) containing 20 ng/mL oncostatin M (OsM) in the absence of epidermal growth factor. 

### 2.7. Preparation of Human iPSC-Derived Endothelial Cells (hiPSC-ECs)

CD31+ cells were prepared from differentiated hiPSCs (Ff-I14). To generate embryoid bodies (Ebs), hiPSCs were seeded onto EZSPHERE (AGC TECHNO GLASS Co., Ltd., Shizuoka, Japan). Approximately 3 × 10^5^ hiPSC cells/mL were cultured in the StemFit AK03N medium containing 2 ng/mL BMP4 and 10 µM Y27632 in the absence of component C (Ajinomoto, Co., Ltd., Tokyo, Japan) (day 0). On day 1, 9 ng/mL BMP4, 10 ng/mL bFGF, and 6 ng/mL Activin A (R&D Systems, Inc., Minneapolis, MN, USA) were added to the medium. From days 2 to 7, Ebs were cultured in a single-use bioreactor and magnetic stirrer (ABLE Corporation & Biott Corporation, Tokyo, Japan). On day 2, the medium was supplemented with 9 ng/mL BMP4, 10 ng/mL bFGF, and 6 ng/mL Activin A (R&D Systems, Inc., Minneapolis, MN, USA), and removed on day 4. On day 4, the medium was supplemented with 25 ng/mL vascular endothelial growth factor (VEGF) (R&D Systems, Inc., Minneapolis, MN, USA) and 8 ng/mL bFGF, and removed on day 7. On day 7, Ebs were enzymatically dissociated and subjected to MACS (Miltenyi Biotec Inc., Tokyo, Japan) to separate CD31+ cells. CD31+ cells were maintained in EGM2 (Lonza, Inc., Basel, Switzerland) on collagen-type IV-coated tissue culture dishes. They were passaged every two days until being harvested for cryopreservation on day 13.

### 2.8. In Vivo Evaluation on the Viability of the Recellularized Scaffold Implanted under the Renal Capsule of Pigs

The decellularized liver scaffold was recellularized with hiPSC-HLCs and human mesenchymal stem cells (hMSCs) as co-seeding MSCs with primary hepatocytes into a decellularized scaffold, effectively maintaining hepatocyte function [9]. Recellularization was performed over 4 d in a sterile closed circumfusion bioreactor containing HCM (Lonza, Inc., Basel, Switzerland) supplemented with 20 ng/mL OsM (R&D Systems, Inc., Minneapolis, MN, USA), 25 ng/mL VEGF (R&D Systems, Inc., Minneapolis, MN, USA), 8 ng/mL bFGF (R&D Systems, Inc., Minneapolis, MN, USA), and antibiotic-antimycotic (Thermo Fisher Scientific K.K., Tokyo, Japan). The bioreactor consists of a gas-permeable membrane at the top and bottom to maintain appropriate oxygen and carbon dioxide concentrations. Several tubes penetrate the membrane, allowing the culture medium to be exchanged and sampled.

Approximately 5.0 × 10^8^ hiPSC-HLCs and 5.0 × 10^7^ hMSCs (Lonza, Inc., PT-2501, Basel, Switzerland) were co-seeded with the same culture medium through the IVC in four subsequent injections at 2 h intervals. The medium was continuously perfused at a rate of 20 mL/min for 4 d. The bioreactor was set in a humidified incubator at 37 °C and 5% CO_2_. The perfusion medium was changed daily. The recellularized scaffold was cut into approximately 1.0 cm-sized pieces and implanted under the porcine renal capsule. Immunosuppressive drugs were administered through gastrostomy after surgery (Figure S2A). The pig was euthanized on postoperative day 14, and the implanted scaffolds were analyzed.

### 2.9. In Vivo Evaluation of the Recellularized Scaffold by the Transplantation via Vascular Anastomosis

The decellularized scaffold was recellularized with the same medium used for recellularization in the implantation into the renal capsule. Approximately 5.0 × 10^8^ hiPSC-HLCs were seeded on the 1st day through the IVC using four subsequent injections at 2 h intervals. Approximately 2.5 × 10^7^ hiPSC-ECs were also seeded on days 1–2 through the IVC using three subsequent injections at 2 h intervals. The medium was continuously perfused at a rate of 20 mL/min. Following 48 h of continuous perfusion of the medium, the flow direction was changed from the IVC to the PV at a rate of 10 mL/min to endothelialize both the portal and venous vasculatures of the scaffold. Thereafter, an additional 2.5 × 10^7^ hiPSC-ECs were seeded in the same manner. The bioreactor was set in a humidified incubator at 37 °C and 5% CO_2_. The perfusion medium was changed daily, and the medium was collected as a sample.

Prior to transplantation, a splenectomy and 60% hepatectomy were performed on the immunosuppressive pig by middle hepatic vein resection using Glisson’s bundle processing. The recellularized scaffold was placed on the caudal side of the remnant liver. Heparinization was performed until the activated coagulation time exceeded 200 s before vascular anastomosis. The preserved PV and IVC in the scaffold were end-to-end anastomosed to the recipient’s PV and IVC, respectively. Following confirmation of blood circulation to the scaffold, the preserved hepatic artery in the scaffold was end-to-end anastomosed to the recipient’s splenic artery in the same manner. A PV catheter was inserted through the splenic vein for angiography using an iodinated intravenous contrast agent (Omnipaque 350, DAIICHI SANKYO Co. Ltd., Tokyo, Japan). Finally, a gastrostomy was performed to administer immunosuppressive drugs (steroids, tacrolimus, and mycophenolic acid). Anticoagulation therapy with continuous intravenous heparin infusion through an intraportal catheter was also administered to prevent thrombosis. The medication contents and methods are shown in Figure S2B. Blood was analyzed every 3 d after the operation. The dose of tacrolimus was adjusted to 6–8 mg/mL according to the serum blood level. Contrast-enhanced computed tomography was performed on postoperative day 14. The pig was euthanized on postoperative day 28, and the transplanted scaffolds were analyzed.

### 2.10. Histological Analysis

Tissue samples were fixed with 4% paraformaldehyde in PBS. The fixed samples were paraffin-embedded and subsequently sectioned in 0.5–1 μm thickness. Sections were stained with hematoxylin and eosin (H and E) according to standard histological techniques.

### 2.11. Immunostaining

Cultured hiPSC-HLCs were fixed with 5% dimethyl sulfoxide in methanol and subsequently blocked with 5% FBS in PBS. Fixed HLCs were stained with primary antibodies, including anti-human ALB (16475-1-AP, Proteintech Inc., Rosemont, IL, USA), SOX17 (ab33101, Abcam Inc., Cambridge, UK), and alpha-fetoprotein (AFP; ab133617, Abcam Inc., Cambridge, UK) overnight at 4 °C. The nuclei were counterstained with 4‘,6-diamidino-2-phenylindole (DAPI; D1306, Thermo Fisher Scientific K.K., Tokyo, Japan). Cultured hiPSC-ECs were fixed in the same manner. The fixed ECs were stained with anti-human CD31 (M0823, Agilent) overnight at 4 °C. Nuclei were counterstained with DAPI. In the immunostaining of tissues, fixed samples were deparaffinized and rehydrated, and the antigens were retrieved. For the immunostaining of the decellularized tissues, the primary antibodies used were anti-fibronectin (ab23751; Abcam Inc., Cambridge, UK) and anti-laminin (ab11575; Abcam Inc., Cambridge, UK). For the implanted scaffold under the renal capsule, the primary antibodies used included anti-human ALB (A6684; Sigma-Aldrich Co., St. Louis, MO USA), pig cross-reactive anti-human CD31 (ab28364, Abcam Inc., Cambridge, UK), CYP3A4 (ab231816, Abcam Inc., Cambridge, UK), and anti-CD44 (ab157107, Abcam Inc., Cambridge, UK). For the transplanted scaffold via vascular anastomosis, the primary antibodies used included anti-human ALB (A16475-1, Proteintech Inc., Rosemont, IL, USA), anti-human CD31 (M0823, Agilent, Santa Clara, CA, USA), and pig cross-reactive anti-human CD31 (ab28364, Abcam Inc., Cambridge, UK). The secondary antibodies used were goat anti-rabbit IgG Alexa Fluor 488 (ab96899, Abcam Inc., Cambridge, UK), donkey anti-mouse IgG Alexa Fluor 488 (ab150105, Abcam Inc., Cambridge, UK), and goat anti-mouse IgG2a Alexa Fluor 555 (A21137, Invitrogen Inc., Carlsbad, CA, USA). The samples were stained with a DAPI-containing mounting medium (P36971, Invitrogen Inc., Carlsbad, CA, USA). All samples were imaged using a BZ-X800 fluorescence microscope (Keyence Corp., Osaka, Japan). The ratio of CD31-positive cells to DAPI-positive cells and the ratio of cell-filled area to the surface area of samples were calculated using the Analysis Applications Hybrid Cell Count in the BZ-X series (Keyence Corp., Osaka, Japan).

### 2.12. ALB and Urea Secretion

The culture supernatants, obtained after 24 h incubation, were analyzed using the Human Albumin ELISA Quantitation Set (Bethyl Laboratories, Inc., Montgomery, TX, USA) to determine the levels of albumin secretion. ELISA was performed according to the manufacturer’s instructions. The amount of secreted albumin was calculated based on the standards, followed by normalization of the protein content per well. The protein content was evaluated using the Pierce BCA Protein Assay Kit (Thermo Fisher Scientific K.K., Tokyo, Japan), according to the manufacturer’s instructions.

To investigate the albumin and urea synthesis ability of the scaffolds recellularized with iPSC-HLCs and iPSC-ECs, culture supernatants were extracted from the bioreactor every day. For reference, iPSC-HLCs at a density of 9 × 10^5^ cells/well and iPSC-ECs at a density of 9 × 10^4^ cells/well were cultured in Matrigel-coated (Corning Inc., Painted Post, NY, USA) 24-well multi dish for 4 days. The culture supernatants from each well were sampled daily. Albumin secretion was measured as described above, and the amount of secreted urea was measured using QuantiChrom Urea Assay Kit (BioAssay Systems, LLC, Hayward, CA, USA). Samples of culture supernatant were analyzed as biological replicates (*n* = 3). Each sample was subjected to three technical replicates for each analysis, and the average value was used according to the manufacturer’s instructions. Normalization was performed based on the total cell number and the amount of culture medium.

### 2.13. Measurement of Cytochrome P450 (CYP) 3A4 Activity

The pieces of scaffolds recellularized with iPSC-HLCs were set in a 24-well multidish at the end of the 4-days culture period. They were compared to iPSC-HLCs cultured for the same period in matrigel-coated 24-well multidish seeded at a density of 9 × 10^5^ cells per well. Both samples were preconditioned with 20 µM rifampicin-containing medium for 48 h; the CYP activity was estimated using the P450-Glo™ CYP3A4 Assay Kit (Promega Co., Madison, WI, USA), according to the manufacturer’s instructions. Briefly, after 2 h of incubation in 3 µM luciferin-IPA-containing medium, 25 µL of culture medium from each well was transferred to a 96-well white plate. The luciferin detection reagent (25 µL) was added to each well and mixed; the mixtures were incubated at room temperature for 20 min. Luminescence was measured using a microplate reader (M1000; Tecan Japan Co., Ltd., Kawasaki, Japan). Samples of iPSC-HLCs cultured in scaffolds and dishes were analyzed as biological replicates (*n* = 3). Each sample was subjected to three technical replicates for each analysis, and the average value was used. Normalization was performed based on the total amount of DNA in each well.

### 2.14. CYP-Related RNA Extraction and Quantitative Real-Time PCR

CYP-related RNA expression was assessed to evaluate the metabolic capacity of hiPSC-HLCs after recellularization into scaffolds. For reference, iPSC-HLCs were seeded at a density of 9 × 10^5^ cells/well in a Matrigel-coated 24-well multi dish and cultured for 4 days. Total RNA was extracted using the RNeasy Mini Kit (QIAGEN K.K.), following the manufacturer’s instructions. Complementary DNA was synthesized from 1 μg of total RNA per sample using Prime Script RT Master Mix (Takara Bio Inc., Shiga, Japan) according to the manufacturer’s instructions. Quantitative real-time PCR was performed using SYBR Master Mix (Thermo Fisher Scientific K.K., Tokyo, Japan) and QuantStudio™ 5 Real-Time PCR System (Thermo Fisher Scientific K.K., Tokyo, Japan). The primer list is presented in Table 1. PCR data were analyzed using the comparative CT method. Samples of iPSC-HLCs cultured in scaffolds and in dishes were analyzed as biological replicates (*n* = 3). Each sample was subjected to three technical replicates of each PCR reaction. β-Actin was used as the internal control.

### 2.15. Statistical Analysis

The intergroup differences between two groups were compared using one-way ANOVA. *p* < 0.05 was considered statistically significant. The analyses were performed using SPSS version 26.0 (IBM Co., Armonk, NY, USA).

## 3. Results

### 3.1. Characteristics of the Decellularized Organ-Derived Scaffold

**Figure 1:**
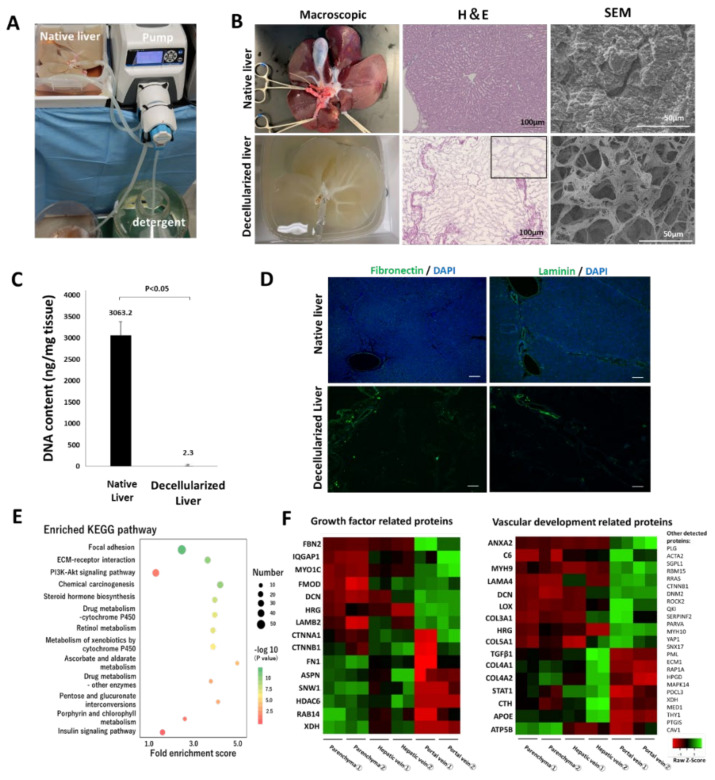
Decellularization of a whole porcine liver and properties of the decellularized scaffold. (**A**) Circulation system for the decellularization. (**B**) Comparison between the native and decellularized livers. Left to right: macroscopic appearance, H&E staining, high-power field SEM in the parenchymal space. In the H&E image, the black square shows a magnified view of the parenchymal space after the decellularization. (**C**) Residual DNA content in the native and decellularized porcine liver (mean ± SD, *n* = 4). (**D**) Immunostaining for the native and decellularized liver using anti-fibronectin and anti-laminin antibodies. Nuclei were counterstained with DAPI. Scale bar = 100 µm. (**E**) Kyoto Encyclopedia of Genes and Genomes (KEGG) pathway analysis of the top 13 proteins with the greatest fold enrichment. X and Y axes denote the fold enrichment score on the KEGG pathway and the term of analyzed pathway, respectively. For the colored dots, the differences in color and size correpond to those in the *p*-value and the number of proteins assigned to each pathway. (**F**) Gene ontology enrichment analysis on proteins related to cell growth factors and vascular development.

The whole porcine liver was decellularized by freeze-thawing and subsequently sustained perfusion of surfactants and buffer solutions (Figure 1A and Figure S1). The decellularized liver scaffold was miniaturized to an appropriate size, allowing ectopic transplantation and in- and out-flow of blood into organ tracts required for transplantation. H&E staining of the decellularized scaffold showed that the cellular components of the native liver were fully removed after decellularization, whereas three-dimensional structures, including ECMs and micro-vascular networks, were preserved (Figure 1B). SEM images show that the pore size of the microfibrillar network of the preserved ECMs was approximately 10–20 µm, which is an appropriate pore size for cell invasion and migration [23]. The removal of cellular components from the native tissue was confirmed through DNA quantification. After the decellularization of the tissue, the DNA content was significantly reduced by 99.9% (Figure 1C). The immunostaining of the native and decellularized livers showed that the ECM components, including fibronectin and laminin, were retained (Figure 1D). Protein expression related to wound repair and regeneration in the decellularized scaffold was examined by exhaustive proteomic analysis using mass spectroscopy. According to the KEGG pathway enrichment analysis based on proteomic analysis, residual proteins in the decellularized scaffold were associated with cell-cell communication, such as ECM-receptor interactions, and signaling pathways involved in cellular proliferation and survival, such as the PI3K-Akt signaling pathway (Figure 1E). As cell types used for recellularization were vascular endothelial cells and hepatocytes, GO enrichment analysis of the proteins associated with cell growth factors, and vascular development was conducted based on proteomic analysis. ECM-related proteins, such as collagens, fibrillin, fibrinogen, fibronectin, and laminin, were distributed non-homogeneously in the different regions (parenchyma, portal, and venous regions) of the liver lobule (Figure 1F). This result was supported by the positive expression of fibronectin and laminin in the PV region (Figure 1D). The proteomic analysis revealed that the constituent proteins were retained with natural localization in the organ-derived scaffold even after decellularization, which may contribute to cell engraftment and development in the appropriate locations during recellularization. Some intracellular components, such as histone deacetylase, were identified during the analysis. However, the degree of decellularization, as confirmed through the DNA quantification test, was comparable to that of earlier reports [24,25]. The degree of decellularization was acceptable, considering that excessive decellularization is accompanied by the loss of bioactive components in the ECMs [26].

### 3.2. Implantation of the Recellularized Scaffold with hiPSC-HLCs under the Renal Capsule

**Figure 2:**
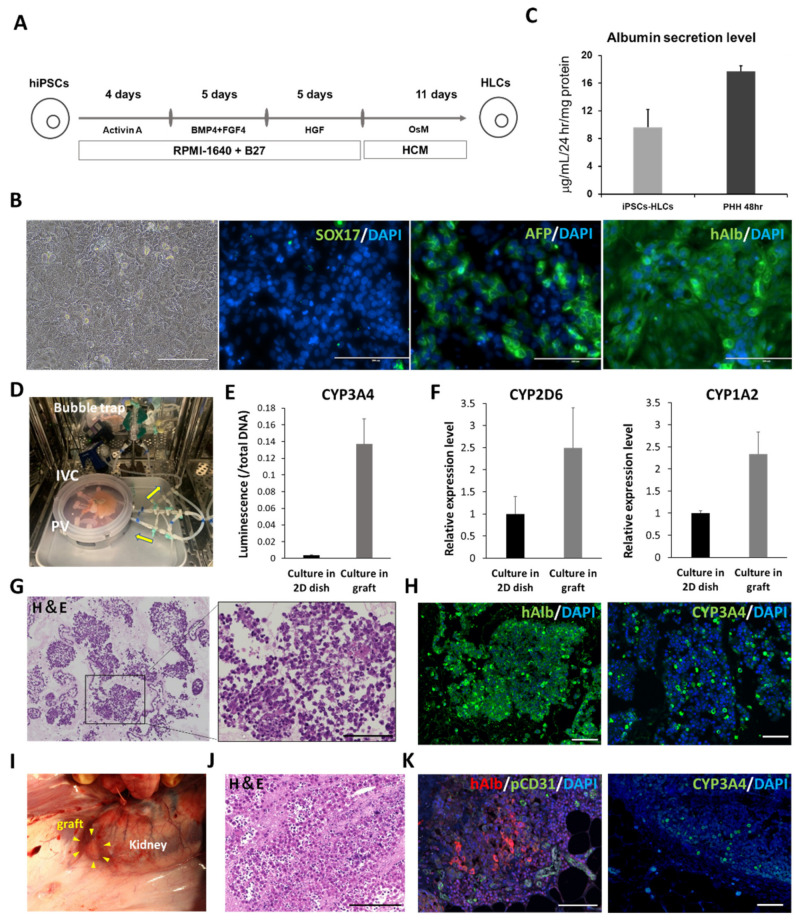
Functional analysis of cultured induced pluripotent stem cell-hepatocyte-like cells (iPSCs-HLCs) on the dish and iPSCs-HLCs seeded into the scaffold. (**A**) Protocol for differentiation induction to hiPSC-HLCs. (**B**) Bright field and immunostaining images of hiPSCs-HLCs at day 25 of differentiation induction. Left to right: bright field, SOX17, human albumin, and AFP. Scale bar = 200 µm. (**C**) Comparison of ALB secretion level between cultured hiPSC-HLCs and primary human hepatocyte (PHH) on dishes. (**D**) Bioreactor for recellularization. (**E**) Comparison of CYP3A4 activity for the cultured hiPSC-HLCs in the scaffold and on the dish (mean ± SD, *n* = 3). (**F**) Comparison of relative CYP-related mRNA expression in the cultured hiPSC-HLCs in the scaffold and on the dish (mean ± SD, *n* = 3) (**G**) H&E staining of the recellularized scaffold. (**H**) Immunostaining images of the recellularized scaffold. Left: human albumin. Right: CYP3A4. Scale bar = 100 µm. (**I**) Renal subepithelial implantation of the recellularized scaffold (**J**) Histological images of the scaffold observed after 14 d of the implantation. Scale bar = 50 µm. (**K**) Immunostaining images of the implanted scaffold. Left: human ALB and pig CD31. Right: CYP3A4. Scale bar = 100 µm.

To evaluate the efficacy of the scaffold as a carrier of hiPSC-HLCs, the functions of the cells seeded in the scaffold were analyzed in vitro and in vivo. hiPSC-HLCs differentiated from iPSCs at day 25 were used for recellularization. The differentiated hiPSC-HLCs presented a paving stone morphology similar to that of primary hepatocytes (Figure 2B). Immunostaining showed albumin-and AFP-positive cells, suggesting that the degree of differentiation from iPSCs to mature HLCs was variable at day 25 in differentiation induction. However, the hiPSC-HLCs were not too immature, because they were negative for SOX17, an endoderm-specific marker [27]. The differentiation of the hiPSC-HLCs was expected to be complete during the circumfusion culture. In general, the ALB secretion capacity of hiPSC-HLCs is significantly lower than that of primary hepatocytes [28,29]. Although the induced hiPSC-HLCs in this study expressed lower albumin secretion capacity than primary hepatocytes, it was considered sufficient for hiPSC-HLCs (Figure 2C). To examine the effect of recellularization of the liver scaffolds with hiPSC-HLCs, the hiPSC-HLCs were cultured in Matrigel-coated dishes and in the decellularized scaffolds using our original bioreactor (Figure 2D). After a 4-day culture, an approximately 40-fold increase in CYP3A4 activity, and upregulation of CYP mRNA expression, were observed in hiPSC-HLCs cultured within the scaffold compared to that in hiPSC-HLCs cultured on dishes (Figure 2E,F). To implant the recellularized scaffolds under the kidney capsule, they were co-seeded with hMSCs and hiPSC-HLCs. H&E staining of the recellularized scaffold showed that the parenchymal space was filled with the seeded cells (Figure 2G). These cells were positive for the expression of the hepatocyte markers, CYP3A4 and ALB (Figure 2H). SEM images also showed that hiPSC-HLCs were embedded in the parenchymal space of the scaffold (Figure S3A). The recellularized scaffold was implanted under the porcine renal capsule (Figure 2I). H&E staining showed numerous transplanted cells at the edge of the scaffold and abundant neovascularization extending from the renal capsule within the scaffold (Figure 2J and Figure S4A). Human ALB-positive cells and CYP3A4-positive cells were detected in the areas where micro-vessel-like structures with CD31-positive cells were observed (Figure 2K). In addition, human-specific Ki-67-positive cells were observed in the scaffold, which suggested the proliferative potential of the implanted human cells (Figure S5A). Sufficient blood would be supplied to the hiPSC-HLCs in the scaffold through the neo-vasculature, in addition to that through the simple diffusion of nutrients and oxygen, to ensure their long-term survival. A small number of human CD44-positive cells were observed in the recellularized scaffold before and after implantation (Figure S5B,C).

### 3.3. Recellularization Scaffold with hiPSC-HLCs and hiPS-ECs

**Figure 3:**
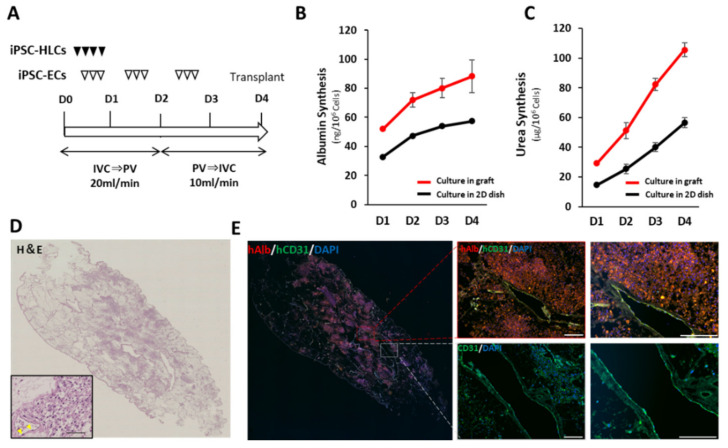
Recellularization of scaffold with hiPSC-HLCs and hiPSC-ECs. (**A**) Protocol for recellularization with hiPSC-HLCs and hiPSC-ECs. (**B**) Accumulative value of ALB secreted from the hiPSC-HLCs into the supernatants of culture medium collected from in-graft and on-dish cultures (mean ± SD, *n* = 3). Values were normalized based on the number of cells used in the culture. (**C**) Accumulative value of urea secreted from iPSC-HLCs in the supernatants of culture medium collected from in-graft and on-dish cultures (mean ± SD, *n* = 3). Values were normalized based on the number of cells used in the culture. (**D**) H&E staining of the recellularized scaffolds sections. A magnified view is shown in the lower left frame. Scale bar = 100 μm. Yellow arrow heads indicate the engrafted hiPSC-ECs on the vessel wall. (**E**) Co-immunostaining images of human ALB and human CD31 counterstained with DAPI. Scale bar = 100 µm.

As the decellularized scaffold maintains a native vascular network structure, it can receive sufficient blood supply from a recipient via vascular anastomosis. Here, the feasibility of in vivo transplantation of a scaffold filled with iPSC-HLCs using such a surgical method was investigated. To achieve transplantation with vascular anastomosis, endothelialization of the vessel lumen inside the scaffold is required to prevent thrombus formation and extravascular leakage of blood [30]. To assess the effect of prior vascular endothelialization before transplantation, we performed angiography on decellularized porcine liver fragments with and without vascular endothelialization using human umbilical vein endothelial cells (HUVEC) (Figure S6A). The contrast agent immediately leaked out of the vessels without endothelialization (Figure S6B); prior endothelialization enabled retaining the contrast agent along the vessels. Therefore, prior vascular endothelialization was necessary (Figure S6C). The scaffold for transplantation was endothelialized using hiPSC-ECs. The protocol for differentiation induction of hiPSC-ECs is shown in Figure S7A. The differentiated hiPSC-ECs had a cobblestone-like shape and were CD31-positive (Figure S7B,C), which was compatible with primary vascular endothelial cells [31]. The ratio of CD31-positive/DAPI-positive cells confirmed that a highly purified endothelial-like cell population was induced (Figure S7D). Recellularization occurred over a period of 4 days (Figure 3A). Seeding efficiency was estimated using the circulating culture medium in the bioreactor after seeding of the hiPSC-HLCs and hiPSC-ECs on day 1. On average, 2.3 × 10^5^ cells were present in the medium, indicating that more than 99.5% of the seeded cells were engrafted in the scaffold. Accumulation of secreted human ALB and urea in the culture medium was examined and compared to that in 2D culture conditions, after normalization for the number of cells (Figure 3B,C). The synthesis of both proteins was promoted approximately 2-fold in the engrafted hiPSC-HLCs, compared to that in cells cultured in a dish. Engraftment and the maintenance of the hiPSC-ECs and hiPSC-HLCs were confirmed using the H&E and immunostaining images of the recellularized graft at the end of the 4-day culture (Figure 3D,E). They indicated that 25−38% of the surface area of recellularized tissue sections was filled with the seeded hiPSC-HLCs. Co-immunostaining with human ALB and human CD31 demonstrated that the hiPSC-HLCs were firmly seeded in the parenchymal region and that the vascular endothelial cells were continuously engrafted in the vascular lumen from the peripheral vessels to the larger vessels. The engraftment of the hiPSC-ECs into the vessel wall was observed in the SEM images (Figure S3B).

### 3.4. Heterotopic Transplantation of the Recellularized Scaffold with the hiPSC-HLCs via Vascular Anastomosis

**Figure 4:**
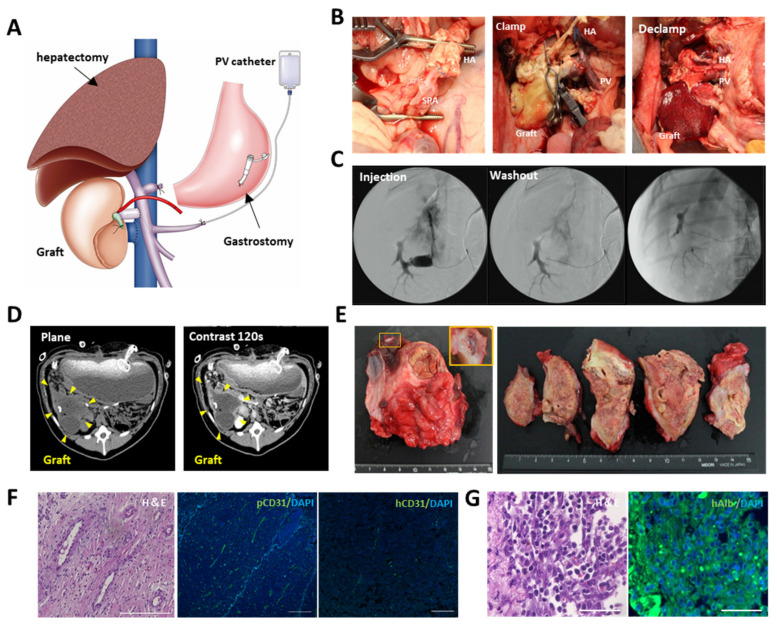
Transplantation of the engineered liver grafts by vascular anastomosis. (**A**) Schema for transplantation of the recellularized scaffold. Partial (60%) hepatectomy, intraportal infusion catheter, and gastrostomy were conducted during surgery. (**B**) Macroscopic images of the transplantation procedure. Left: anastomosis between hepatic artery of the scaffold and the splenic artery of the recipient. Middle: the scaffold before blood perfusion. Right: the scaffold after blood perfusion. (**C**) Intraoperative angiography through the intraportal infusion catheter. (**D**) Contrast enhanced computer tomography (CT) images of the transplanted graft at postoperative day 14. (**E**) Macroscopic images of the transplanted graft at postoperative day 28. (**F**) H&E images of the parenchymal space of the transplanted scaffold and immunohistological images of CD31 stained by pig cross-reactive anti human CD31 or anti-human CD31. Immunohistological images were counterstained with DAPI. Scale bar = 100 µm. (**G**) (left) H&E staining and (right) immunostaining of anti-human albumin at the edge of the scaffold. Scale bar = 50 µm.

The recellularized scaffold was transplanted to the caudal side of the 60% hepatectomized remnant liver via portal and venous anastomosis (Figure 4A). The hepatic artery of the scaffold was anastomosed to the recipient’s splenic artery (Figure 4B). Following anastomosis, vessel clumps were released. Blood flow immediately circulated throughout the scaffold, and the scaffold turned red. The leakage of blood from the surface of the scaffold and the anastomotic area was prevented by the structural strength of the scaffold. Following surgery, angiography was performed through the intraportal catheter inserted into the splenic vein. The contrast medium was washed out over time, indicating that continuous blood flow was maintained after surgery (Figure 4C). There were no signs of infection or intra-abdominal bleeding during the perioperative period. Although the structural strength of acellular scaffolds has been an issue [32], dynamic CT on postoperative day 14 showed that the transplanted scaffold maintained its morphology without atrophy. The portal inflow of the graft was barely visible; however, contrast enhancement was observed at the edge of the graft (Figure 4D), suggesting neovascularization from the area surrounding the graft, as observed in the implantation to the renal capsule. Recipient pigs were euthanized on postoperative day 28. The inside of the transplanted scaffold was reddish, indicating that the blood supply was maintained (Figure 4E). Delayed fibrous stenosis was observed at the anastomotic site, although this was not confirmed at the time of postoperative angiography. As the patency of blood vessels was maintained, stenosis was considered to be caused by reduced blood supply through the anastomosis. Despite the reduced blood flow, H&E staining of the inside of the scaffold showed abundant vascular-like structures around the edge of the scaffold (Figure 4F and Figure S5B). These structures were stained with pig cross-reactive anti-human CD31 antibody, and not with human-specific CD31 antibody (Figure 4F). The hiPSC-ECs were considered dead by postoperative day 28 because of inadequate blood flow caused by anastomotic stenosis. Considering that hiPSC-ECs were firmly engrafted to the vascular lumen before transplantation, and that fibrous narrowing of the vessels should occur gradually, it was assumed that the cells were gradually inactivated and dead until pig-derived neovascularization from the surrounding area occurred. Human ALB-positive cells were observed around the edge of the scaffold, where the blood supply was well maintained, but not in the center of the scaffold (Figure 4G and Figure S5B). The remnant liver tissue was analyzed for the possibility of migration and engraftment of iPSC-derived cells to other organs, through immunostaining with anti-human CD31 or anti-human albumin antibody. No iPSC-derived cells were observed in the remnant liver (Figure S8).

## 4. Discussion

IPSCs can be created from patient tissues, then differentiated into a variety of cell types. Differentiated iPSC-derived cells have the same genetic makeup as donor patients, making the cells an ideal cell source for ex vivo disease models and pharmacokinetic studies and to support end-stage organ failure. Recently, research on regenerative medicine using iPSC-derived cells has markedly progressed through the realization of an ample supply of these cells [33]. Considering future use of these cells in disease research and transplantation, the aim of this study was to demonstrate that an organ-derived scaffold is highly suitable as a carrier of a large number of hiPSC-derived cells and that the scaffold can be applied to both ex vivo and in vivo applications.

The essential elements of a cell carrier are scaffolds and signaling molecules. The ideal platform should consist of three-dimensional microenvironments that structurally mimic native tissues and allow a supply of oxygen and nutrients to seeded cells and discharge cell-derived metabolites. It is also necessary for the platform to contain important signaling molecules required for cell proliferation, differentiation, and functional expression. It has been challenging to develop synthetic materials that fulfill these requirements; however, the use of decellularization techniques for organs has provided an avenue to meet these requirements.

Although various methods for decellularization have been reported, no unified protocol has been established [34]. As confirmed by SEM and histological staining, the combination of freeze-thawing, and a series of detergents (SDS, Triton, CHAPS), were adopted to remove cellular components without disrupting the microstructures of the native liver tissue. The interactions between cells and ECMs are induced by signal transduction through cell membrane receptors. For example, at desmosomes, focal adhesion kinase for the regulation of cell proliferation and the PI3-kinase Akt pathway for control of cell survival are activated [35,36]. KEGG pathway analysis of the decellularized scaffold showed that functional proteins were significantly maintained in the scaffold, which is beneficial for interactions between the scaffold and seeded cells and for the expression of cellular functions. Wang et al. cultured iPSC-HLCs using a liver-derived scaffold, a polylactic acid-based scaffold, and culture dishes to evaluate their function [20]. The results showed that the cells cultured on the liver-derived scaffold showed higher P450 mRNA expression and metabolic enzyme activities than those cultured on the polylactic acid-based scaffold or culture dishes. They concluded that both multidimensional growth and appropriate matrix biochemical complexity contribute to maturation and functional expression in iPSC-HLCs. These findings support the utility and eligibility of using prepared decellularized scaffolds in iPSC-HLC culture.

Notably, a variety of signaling molecules for cellular processes are localized, depending on each part of the scaffolds, even after decellularization. Proteomic analysis showed that fibromodulin, a promoter of angiogenesis, was localized in the hepatic vein and PV regions. Fibromodulin (FMOD) activates quiescent endothelial cells by increasing the expression of collagen I and III, VEGF, and angiopoietin-2, which stimulate endothelial cell growth, migration, and capillary network formation [37]. In addition to preserved vascular structures after decellularization, it has been reported that new vascular structures at the sinusoidal level were formed under appropriate culture conditions [38]. Thus, preserving the localization of signaling molecules, such as FMOD, in decellularized scaffolds is a key advantage for endothelialization by seeding endothelial cells through retained vessels. In this study, iPSC-derived endothelial cells were successfully engrafted in the lumens of blood vessels, from large vessels to those of the periphery, which is expected to lead to the further development of microvascular structures. In addition, hepatocytes express different functions in a zone-specific manner within lobules [39], and their zonation is reconstituted by changing the ratio of ECM components [40]. These reports suggest that investigation of organ skeletal components in which zone-specific ECM structures are preserved may lead to further understanding of metabolic zoning.

Organ-derived scaffolds serve as a platform for effectively reproducing in vivo and in vitro cellular responses and behaviors by exerting signaling molecules for various cellular processes in appropriate locations. To further reproduce the physiological environments of native organs, we constructed a circumfusion bioreactor that supports human-scale three-dimensional culture by refluxing the medium into scaffolds via a preserved vascular network. After seeding a large number of hiPSC-HLCs using this culture model, it was confirmed that hiPSC-HLCs were appropriately engrafted in the parenchymal space of the scaffold. In addition, increases in ALB production and CYP activity and upregulation of CYP-related mRNA expression were observed compared to traditional two-dimensional cultures. Takeishi et al. reported that the circumfusion culture using a rat liver scaffold promoted the differentiation of liver constituent cells, such as hepatocytes, cholangiocytes, and vascular endothelial cells [14]. Thus, the importance of three-dimensional culture using organ scaffolds is expected to increase for ex vivo analyses that reflect biological responses in vivo.

We evaluated whether the organ scaffold could be directly used as a carrier for iPSC-derived cells in vivo using large animals. The concept of cell-filling of scaffolds for transplantation has attracted attention as an innovative solution to the shortage of donated organs. However, only a few reports exist on the transplantation of recellularized scaffolds due to technical difficulties. In particular, reports on the use of human iPSC-derived cells have been limited to transplantation for a few days in small animals [14,41]. Although no standard immunosuppressed large-animal model has been established, we applied an immunosuppressive regimen to thymectomized micro-miniature pigs based on perioperative management used in ABO-incompatible liver transplantation in humans [42]. In applying iPSC-derived cells to transplantation therapy, cell viability after transplantation is one of the most critical issues. In the present study, hiPSC-HLCs seeded in the organ scaffold and implanted under the renal capsule were nourished by abundant host neovascularization induced within the scaffold. The survival of the hiPS-HLCs was confirmed for two weeks despite xeno-implantation. In addition, engraftment of the hiPSC-HLCs was maintained for approximately a month after transplantation via vascular anastomosis. Thrombus formation has been a concern in the use of decellularized blood vessels, and either endothelialization or chemical modification of scaffolds, such as heparin polymerization, have been devised [30]. We performed endothelialization by seeding hiPSC-ECs into the scaffold prior to transplantation by vascular anastomosis, resulting in angiographic confirmation of active blood flow along the vasculature. However, the long-term maintenance of sufficient blood flow into the scaffold was difficult because of intimal thickening at the anastomosis site. Numerous attempts have been made to use decellularized vascular grafts for vascular bypass; however, the degree of intimal thickening has been reported to be slight [43]. Furthermore, reports of long-term observations after the transplantation of decellularized organ scaffolds via vascular anastomosis are lacking. Therefore, the problem of intimal thickening observed in the present study can serve as an important reference for the transplantation of decellularized organ scaffolds. The mechanism underlying intimal thickening is thought to be the damage to vascular endothelial cells associated with transplantation, the intravascular infiltration of blood macrophages, and the migration of smooth muscle-like cells into the intima and their synthesis of ECM. The severe intimal thickening observed in the present study might be related to the extensive surgical invasiveness associated with transplantation of the scaffold and the immunological mechanisms associated with xenotransplantation. Although there is no definitive treatment, various new drugs such as angiotensin2 receptor blockers [44] and p38 MAPK inhibitors [45] and immunosuppressive agents, such as sirolimus, have been studied [46]. The application of these drugs to the transplantation of scaffolds via vascular anastomosis may allow long-term vessel patency and maintenance of transplanted cells.

In this study, we revealed that the decellularized organ scaffold is a promising carrier that assists the functional maintenance of iPS cell-derived cells by mimicking their biological environment in vivo and that the scaffold has high clinical extrapolation potential for surgical transplantation into large animals under immunosuppressive therapy. The use of decellularized scaffolds and iPS cell-derived cells has high applicability for ex vivo and in vivo analyses. The limitation of this study is that we were unable to seed all cell types that constitute the liver simultaneously. As current technology does not permit mass culturing of all iPSC-derived cell lines that constitute an organ, only iPSC-HLCs and ECs were used in this study. We demonstrated that long-term survival of hepatocytes is achievable. However, for further long-term maintenance and functional analysis, it is necessary to establish a bile excretion pathway by co-culturing epithelial cells of the bile duct along with the non-parenchymal cells responsible for organ homeostasis. Circumfusion culture with decellularized organs allows the seeding of multiple cell types and accommodates a large number of cells; therefore, this culture system could be further improved with the advancement of technology to induce differentiation in iPS cells effectively. The realization of a human-scale three-dimensional carrier containing a large number of iPSC-derived cells will contribute to the elucidation of the pathogenesis of diseases on an organ scale and provide help to the solution of the global shortage of donated organs. The use of decellularized organ scaffolds is expected to become a fundamental technology for regenerative medicine in the future.

## Figures and Tables

**Table 1:** Primers used for real-time PCR.

Transcription	Prime Sequences	
CYP1A2	Forward:	TCGTAAACCAGTGGCAGGT
	Reverse:	GGTCAGGTCGACTTTCACG
CYP2D6	Forward:	ACACCATACTGCTTCGACCA
	Reverse:	ACTGCTCCAGCGACTTCTTG
*β*-Actin	Forward:	GAGCGCGGCTACAGCTT
	Reverse:	TCCTTAATGTCACGCACGATTT

## Data Availability

The data of the current study are available from the corresponding author upon reasonable request.

## Supplementary Materials

The following are available online at https://www.mdpi.com/article/10.3390/cells11081258/s1, Figure S1: The protocol for decellularization of a porcine liver. Figure S2: Postoperative medication protocol. Figure S3: Scanning electron microscopy (SEM) image of the scaffold recellularized with human in-duced pluripotent stem cell derived cells. Figure S4: Hematoxylin and Eosin (H&E) images of implanted or transplanted scaffolds. Figure S5: Immunostaining images of a recellularized scaffold used for implantation under the renal capsule. Figure S6: Efficacy of vascular endothelialization., Figure S7: Preparation of Human iPSC-Derived Endothelial Cells (hiPSC-ECs). Figure S8: Absence of iPS-derived cells in the remnant porcine liver.
