# Supplementary material for: Decellularized Organ-Derived Scaffold Is a Promising Carrier for Human Induced Pluripotent Stem Cells-Derived Hepatocytes
Source: Cells. 2022 Apr 7;11(8):1258. doi: 10.3390/cells11081258 (PMC9025569; doi:10.3390/cells11081258)
Supplement: Supplementary file 1 [file cells-11-01258-s001.zip › cells-1535300-supplementary.pdf]

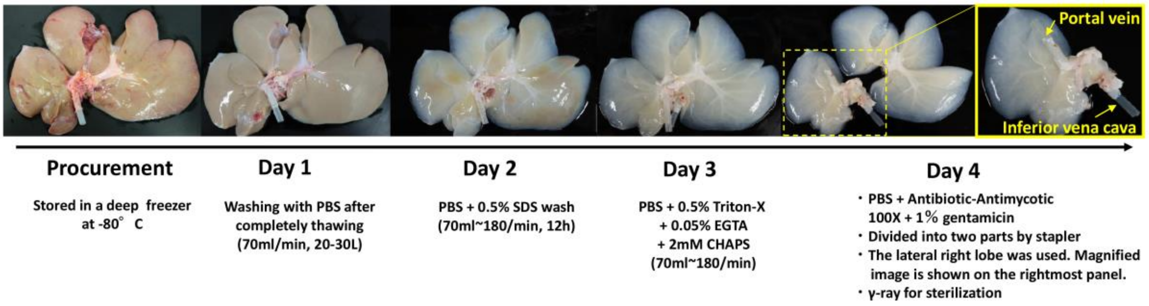

Figure S1: The protocol for decellularization of a porcine liver.

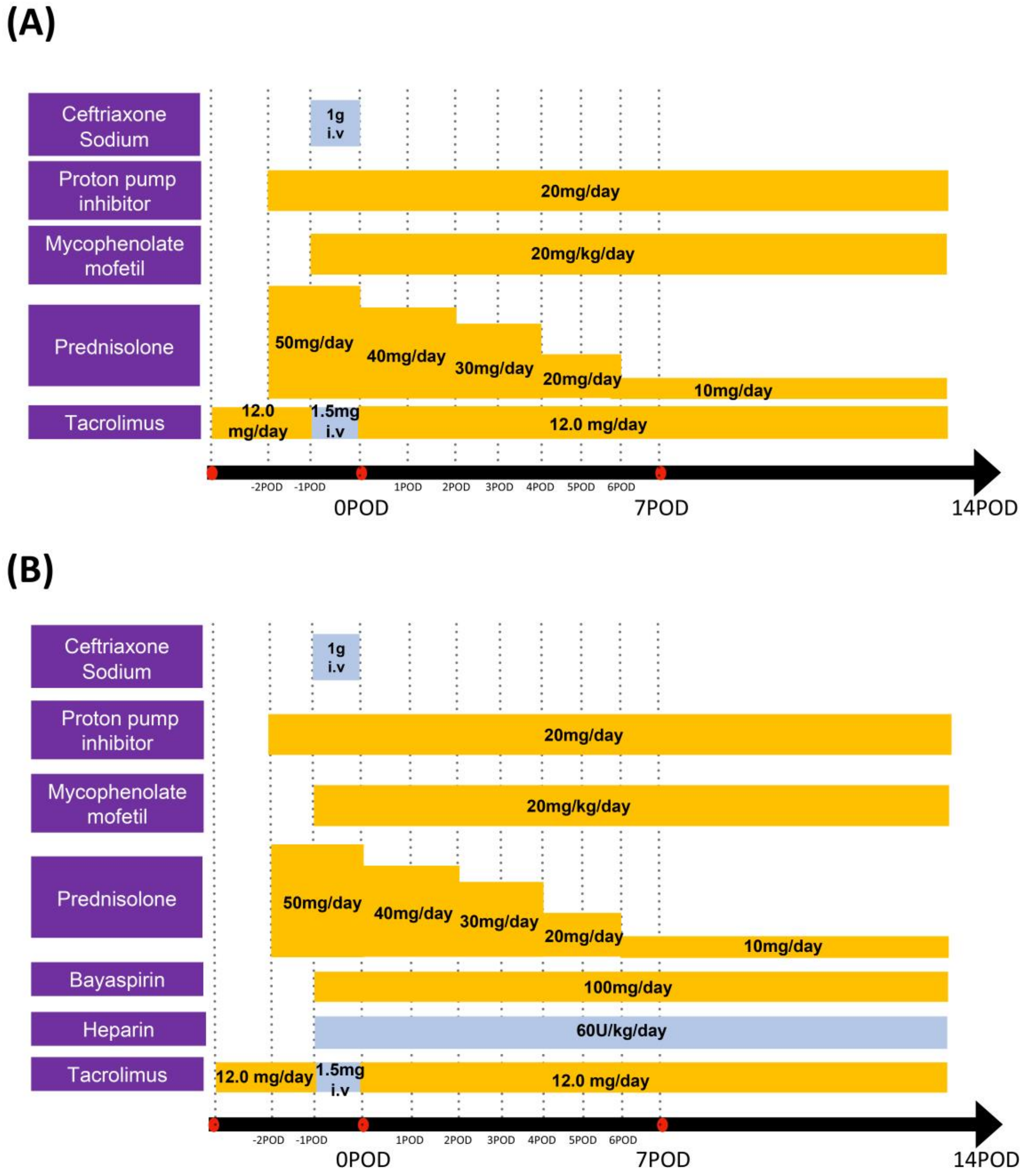

Figure S2: Postoperative medication protocol. (A) Medication protocol after the implantation of the recellularized scaffolds under the renal capsule. (B) Medication protocol after the transplantation of the recellularized scaffolds via vas-cular anastomosis.

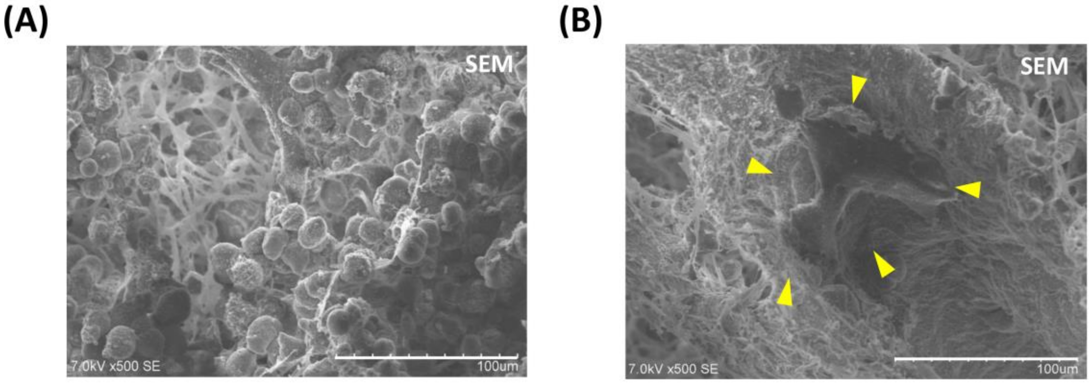

Figure S3: Scanning electron microscopy (SEM) image of the scaffold recellularized with human in-duced pluripotent stem cell derived cells.(A) SEM image of the parenchymal space of the recellularized scaffold. Scale bar = 100 μm. (B) SEM image of the vessel wall coated with hiPSC-ECs. Scale bar = 100 μm.

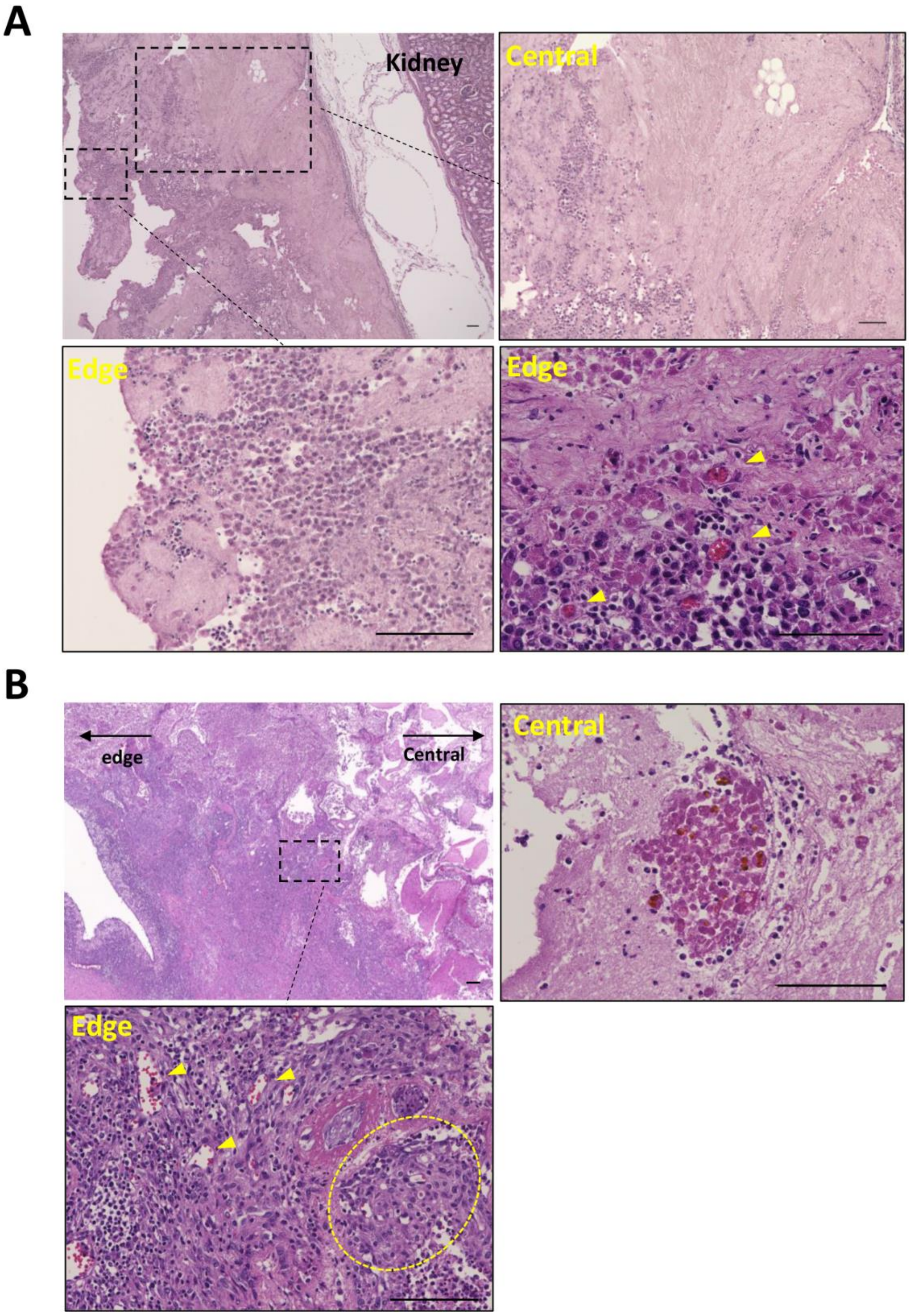

Figure S4: Hematoxylin and Eosin (H&E) images of implanted or transplanted scaffolds. (A) H&E images of recellularized scaffold implanted under the renal capsule at post operative day 14. Yellow arrow heads indicate neovascularization within the scaffold. Red blood cells were observed in the vessel lumen. Scale bar = 100 μm. (B) H&E images of the recellularized scaffold transplanted via vascular anastomosis at post operative day 28. Yellow arrow heads indicate ne-ovascularization within the scaffold. Yellow dotted circles indicate viable hiPSC-HLCs on the edge of scaffold. Red blood cells were observed in the vessel lumen. Scale bar = 100 μm.

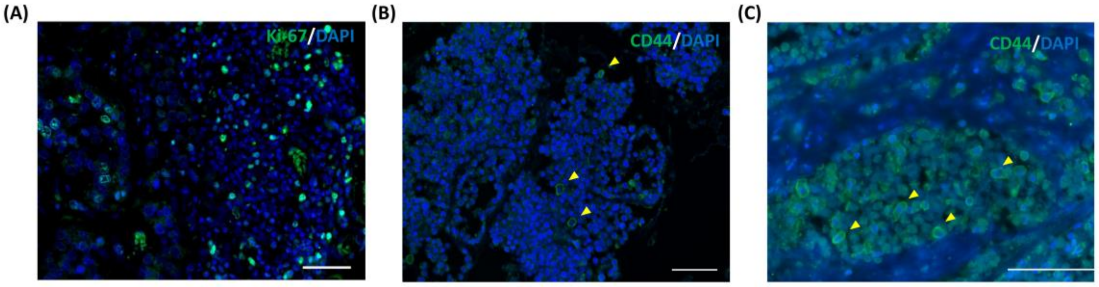

Figure S5: Immunostaining images of a recellularized scaffold used for implantation under the renal capsule. (A) Immunostaining of the recellularized scaffold implanted under the renal capsule, with anti-Ki-67 antibody, at post operative day 14. Scale bar = 100 μm. (B) Immunostaining with anti-CD44 antibody of the recellularized scaffold before implantation. Scale bar = 100 μm. (C) Im-munostaining of the recellularized scaffold implanted under the renal capsule, with anti-CD44 antibody, at post operative day 14. Scale bar = 100 μm.

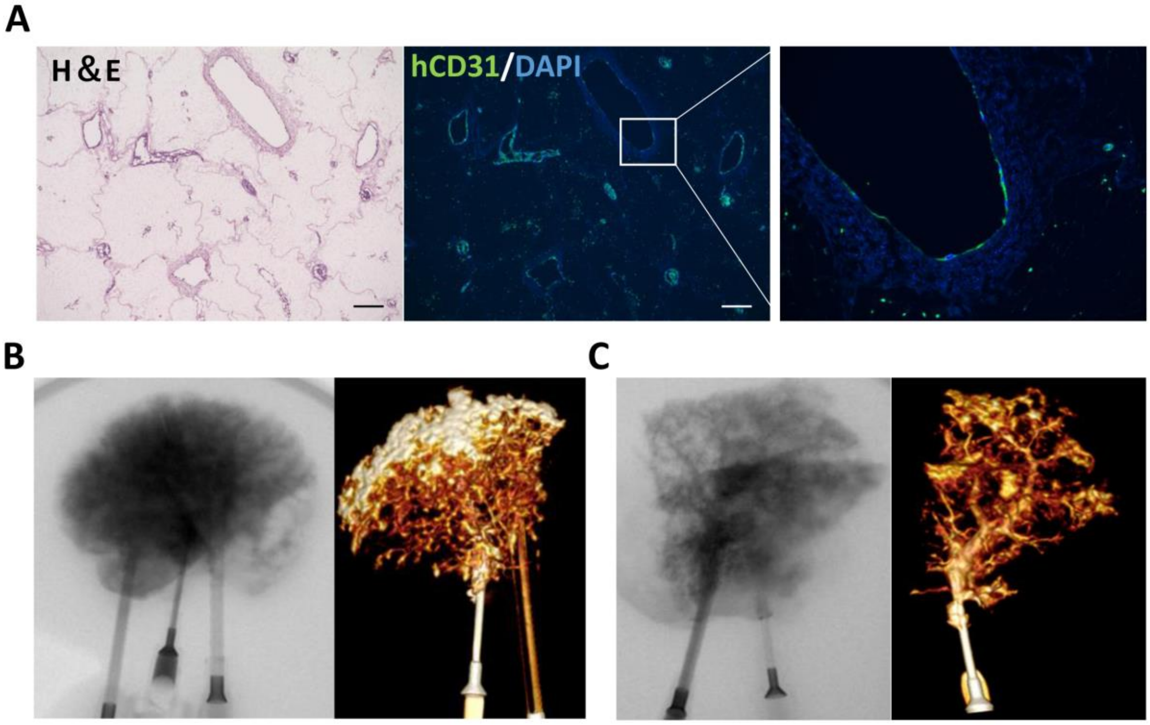

Figure S6: Efficacy of vascular endothelialization.(A) H & E and immunostaining images of the recellularized scaffold using HUVEC. Left: H & E. Middle: immunostaining with anti-CD31. Right: Magnified view of the immunostaining. Scale bar = 100 μm. (B) Angiography of the scaffold without endothelialization. (C) Angiography of the scaffold with endothelialization.

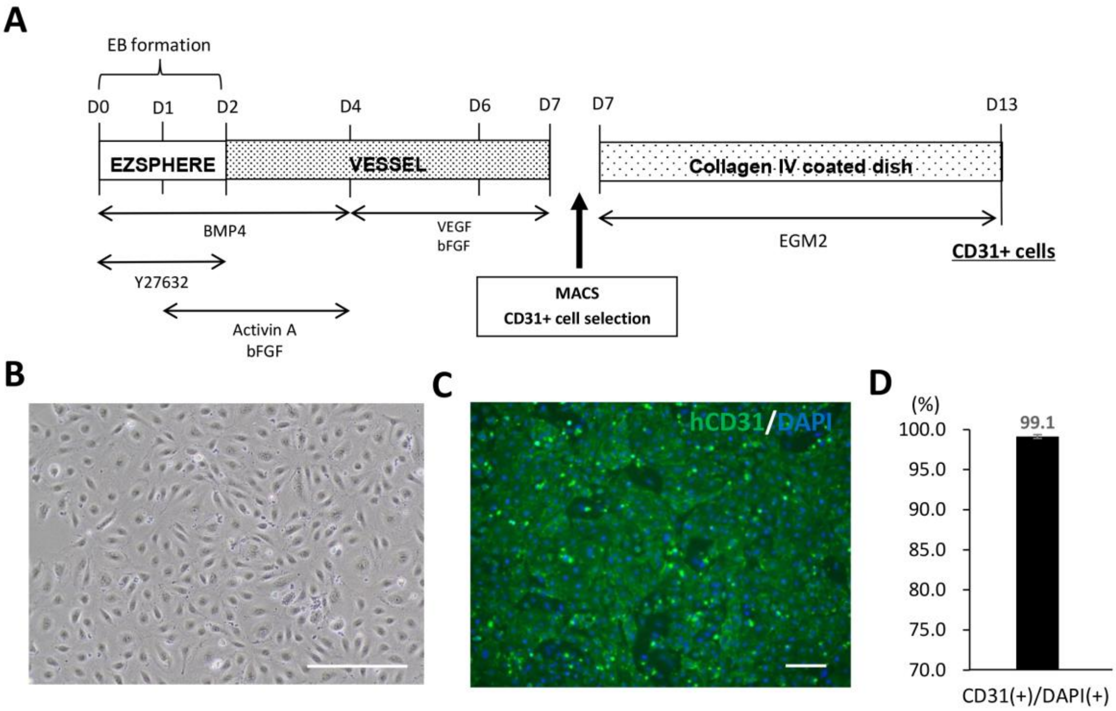

Figure S7. Preparation of Human iPSC-Derived Endothelial Cells (hiPSC-ECs). (A) Protocol for differentiation induction in hiPSC-ECs. (B) Bright field image of the hiPSC-ECs (C) Immunostaining images of CD31 counterstained with DAPI. Scale bar = 100 μm. (D) Average ratio of CD31-positive/DAPI-positive cell in 6 fields of view at 10x magnification.

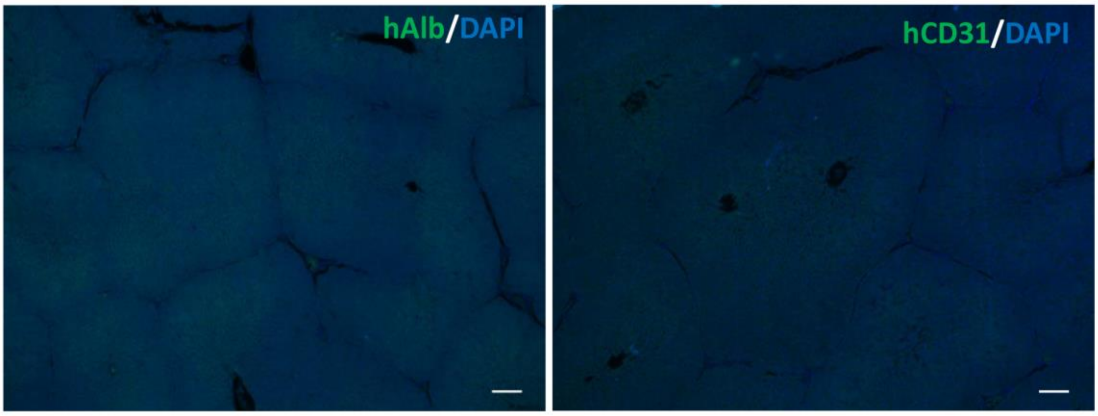

Figure S8. Absence of iPS-derived cells in the remnant porcine liver.Left: Immunostaining images of human anti-albumin counterstained with DAPI. Scale bar = 100 μm. Right: Immunostaining images of human anti-CD31 counterstained with DAPI. Scale bar = 100 μm.
